# Transcutaneous Vagal Nerve Stimulation (tVNS): a new neuromodulation tool in healthy humans?

**DOI:** 10.3389/fpsyg.2015.00102

**Published:** 2015-02-10

**Authors:** Jelle W. R. Van Leusden, Roberta Sellaro, Lorenza S. Colzato

**Affiliations:** Cognitive Psychology Unit, Institute for Psychological Research and Leiden Institute for Brain and Cognition, Leiden UniversityLeiden, Netherlands

**Keywords:** tVNS, neuromodulation, cognitive neuroscience, norepinephrine, GABA

## Introduction

**Figure 1:**
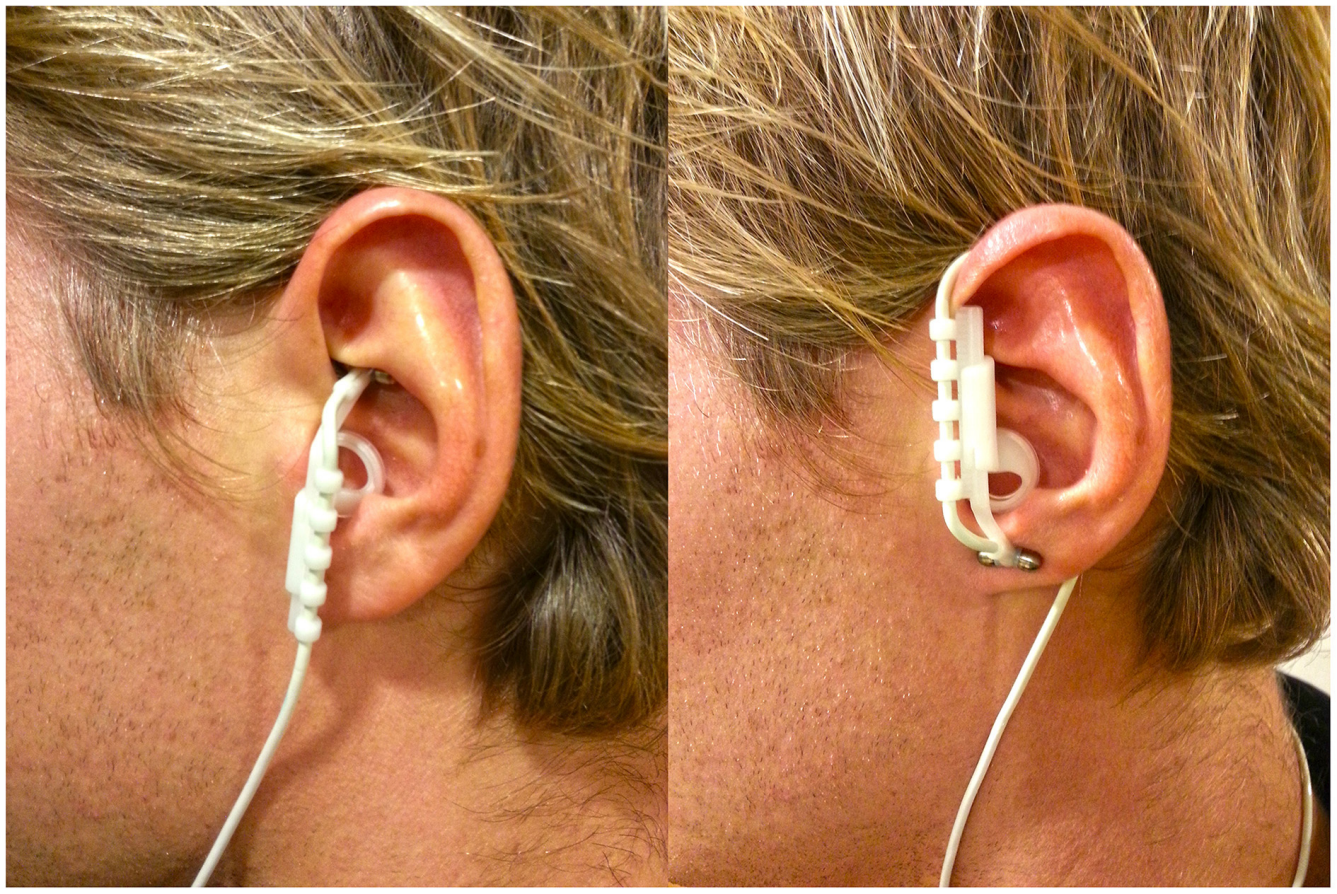
**Positioning of the stimulation electrodes in the active (left) and in the sham (right) condition**.

The idea that we can influence neurons with electricity is not new. Earlier this century patients were treated, and still are, with electro convulsive therapy as a treatment for severe depression (Fink, 1984). Fortunately, new devices were invented that use electricity to influence neuronal activity in a less invasive way: transcranial magnetic stimulation (TMS), transcranial direct current stimulation (tDCS) and vagus nerve stimulation (VNS). In contrast to imaging techniques, which are only correlational, by means of these techniques it is possible to infer a causal relation between the stimulated neurotransmitter/brain area and a related cognitive function. Recently, Cerbomed (Erlangen, Germany) engineered a noninvasive, transcutaneous (through the skin) VNS device (tVNS) that stimulates the afferent auricular branch of the vagus nerve located medial of the tragus at the entry of the acoustic meatus (Kreuzer et al., 2012). This device has received CE approval as indication that it complies with essential health and safety requirements. Thus, tVNS is safe and accompanied only by minor side effects such as slight pain, burning, tingling, or itching sensation under the electrodes. Nevertheless, as specified in the instructions manual, the use of the device is contraindicated in the case of pregnancy, cardiac diseases, head trauma, alcoholism, migraine, medication or drug use, neurological or psychiatric disorders, metal pieces in the body (pacemaker), active implants such as a cochlear implant, wounds and diseased skin. A number of studies using high intensity tVNS have not found any major side-effects (Kraus et al., 2007; Dietrich et al., 2008). Given that the right vagal nerve has efferent fibers to the heart, tVNS is safe to be performed only in the left ear (Sperling et al., 2010; Kreuzer et al., 2012). Following Kraus et al. (2007), a clever way to create a sham condition using tVNS is by attaching the stimulation electrodes to the center of the left ear lobe, which is known to be free of cutaneous vagal innervation (Peuker and Filler, 2002), see Figure 1. Indeed, a recent functional magnetic resonance imaging (fMRI) study showed that this sham condition produced no activation in the cortex and brain stem (Kraus et al., 2013).

So far, VNS has been used to study cognitive functioning only in patients with epilepsy and major depression (Vonck et al., 2014). However, the focus of the present opinion article is not on clinical populations but on healthy humans and how tVNS may be a useful tool to further investigate the neuromodulation of cognitive processes related to norepinephrine (NE), gamma-aminobutyric acid (GABA) and Acetylcholine (ACh), the three main neurotransmitters targeted by VNS. To this end we discuss a number of NE, GABA, and ACh-related cognitive functions that could be modulated by tVNS. This is by no means an exhaustive list; the aim of this opinion article is rather to point out and highlight some theoretically driven links that may help to improve designing future tVNS studies. So far, the studies discussed below have not yet been investigated in combination with tVNS in healthy humans. However, based on literature that details their relation to NE and GABA functions we argue that these studies will prove fertile for future research.

## tVNS, NE, post-error adjustments, emotional memory and implicit negative attitudes

In rats, it has been demonstrated that VNS leads to an intensity-dependent increase in brain NE in response to stimulation of the left vagus nerve (Roosevelt et al., 2006; Raedt et al., 2011). These increases in NE are transient and return to base-line levels when the stimulation is stopped and the vagus nerve is no longer being stimulated (Roosevelt et al., 2006). This transient increase in NE should not be surprising given the anatomical connections between the vagus nerve and the locus coeruleus (LC), the noradrenergic supply center of the brain (Aston-Jones et al., 1991). A phenomenon related to the LC-NE system is the so-called post-error slowing (PES): people tend to slow down their task performance after they make an error. Particularly, PES is indicated by longer reaction times (RTs) on trials succeeding an error than on trials succeeding a correct response (Rabbitt, 1966). PES has been detected in an extensive range of tasks (Danielmeier and Ullsperger, 2011), comprising the Stroop task (Gehring and Fencsik, 2001), the flanker task (Debener et al., 2005; Krämer et al., 2007) and the Simon task (King et al., 2010; Danielmeier and Ullsperger, 2011). Ullsperger et al. (2010) have suggested that slowing after negative feedback or unpredicted errors is connected to the activity of the neuromodulatory LC-NE system. Very recently, Colzato et al. (2013) have demonstrated that an individual's magnitude of PES is predicted by the DBH5′-ins/del polymorphism—a variation in the DBH gene linked with the synthesis of the enzyme dopamine beta-hydroxylase, which is responsible for the conversion of dopamine into NE. Increased PES was associated with DBH5′-ins/del heterozygotes (linked to average level of plasma DβH activity) in contrast to del/del and ins/ins homozygous individuals (linked to low and high level of plasma DβH activity, respectively).

NE plays an important role not only in PES but also in emotional memory (Chamberlain et al., 2006) and implicit racial attitudes (Terbeck et al., 2012). First, the fact that emotional events are remembered better than neutral events (Cahill and McGaugh, 1998) is linked to the amygdala-hippocampus connections, which are modulated by beta-adrenergic-dependent agents (Strange and Dolan, 2004). Moreover, the supplementation of the beta-adrenergic antagonist propranolol has been found to reduce emotional distraction in working memory (Oei et al., 2010). Second, Terbeck and colleagues have found that propranolol abolished implicit racial bias, as indexed by the racial implicit association test (IAT), without affecting the measure of explicit racial prejudice. Given the relation between the above-mentioned cognitive functions and NE, and between NE release and VNS, we argue that active tVNS, as compared to earlobe sham stimulation, may modulate PES, emotional memory, and implicit racial bias.

## tVNS, GABA, bistable perception and action cascading

VNS seems to increase the levels of free GABA in the cerebrospinal fluid (Ben-Menachem et al., 1995). In epileptic patients receiving VNS for a year, GABA-A receptor density in the hippocampus was significantly increased as compared to controls (Marrosu et al., 2003). Berlau and McGaugh (2006) suggested that the GABAergic system modulates memory consolidation by regulating NE release within the amygdala and the hippocampus. Interestingly, via the LC, the vagus nerve reaches indirectly both the amygdala and the hippocampus, areas involved in the storage of memory (Kraus et al., 2007, 2013). Not surprisingly, Ghacibeh et al. (2006) found after VNS an amelioration of the consolidation process of memory in patients suffering from refractory epilepsy. Besides memory, GABA seems to affect the dynamics of bistable perception (van Loon et al., 2013). Higher GABA concentrations (indexed by magnetic resonance spectroscopy; MRS) in visual cortex and lorazepam administration (pharmacological stimulation of the GABA-A receptor) modulated the dynamics of bistable perception by showing slower perceptual dynamics, as reflected in a reduced number of perceptual switches and a lengthening of percept durations. Moreover, a very recent MRS study showed that superior performance in action cascading was associated with increased concentrations of striatal GABA (Yildiz et al., 2014). Given the relation between GABA and the aforementioned cognitive functions, and between GABA release and VNS, we expect active tVNS, as compared to earlobe sham stimulation, to impact the dynamics of bistable perception and to enhance response selection functions when two actions are executed in succession.

## tVNS, ACh, action coordination and memory encoding

In rats, VNS has been found to attenuate the systemic inflammatory response to endotoxin via ACh release (Borovikova et al., 2000). ACh is one of the principal neurotransmitters in both the central and peripheral nervous systems for a variety of functions, including movements and actions (e.g., Watanabe et al., 1990) and the encoding of stimuli into memory (Bartus et al., 1982). Indeed, animal literature suggest that ACh is responsible for the proper development of action coordination in rats and that it plays an essential role in neural communication in brain networks implicated in visuospatial memory (Bartus et al., 1985). Given the link between ACh and the aforementioned cognitive functions, and between ACh release and VNS, we assume active tVNS, as compared to earlobe sham stimulation, to modulate motor control and memory of object locations.

## tVNS and U-shaped function

tVNS seems to exhibit an inverted U-shaped relationship between stimulation intensity and memory performance. Indeed, Clark et al. (1995) showed that rats who received the intermediate stimulation of 0.4 mA current intensity showed significant better avoidance memory than those in the 0.2, 0.8 mA, or control condition. These results were also confirmed by a follow-up study from the same laboratory in humans suffering from epilepsy (Clark et al., 1999), showing that the intermediate stimulation intensity yielded the best performance in a recognition memory task. This is consistent with the inverted-U shaped Yerkes-Dodson principle (Yerkes and Dodson, 1908) that, although originally advanced to explain performance decrements associated with too high or too low arousal levels with increasing task difficulty, seems to be suitable to account for several other non-linear relationships (MacDonald et al., 2006). That being said, it is reasonable to expect also for all other noradrenergic, GABAergic, cholinergic cognitive functions the same pattern of optimal performance at intermediate stimulation and cognitive decrements in case of too high or too low intensity stimulation.

## Conclusion

tVNS may be a useful tool to further investigate the neuromodulation of cognitive processes related to NE, GABA and ACh, the three main neurotransmitters targeted by VNS. Yet, in healthy humans studies on how tVNS may modulate noradrenergic, GABAergic and cholinergic cognitive functions are lacking. Keeping in mind safety guidelines, we suggest that future studies on tVNS should take into account stimulation intensity to achieve a better theoretical understanding of the effect of tVNS on cognition.

### Conflict of interest statement

The authors declare that the research was conducted in the absence of any commercial or financial relationships that could be construed as a potential conflict of interest.
